# Pacing-Induced Cardiomyopathy in Leadless and Traditional Pacemakers: A Single-Center Retrospective Analysis

**DOI:** 10.7759/cureus.41393

**Published:** 2023-07-05

**Authors:** Khalid Saeed Al-Asad, Adolfo Martinez, Rohan M Prasad, Esosa U Ukponmwan, Zulfiqar Q Baloch, Abbas Ali, John Ip

**Affiliations:** 1 Department of Internal Medicine, Michigan State University, East Lansing, USA; 2 Department of Cardiology, Sparrow Hospital, Lansing, USA; 3 Department of Cardiology, West Virginia University, Morgantown, USA

**Keywords:** cardiac resynchronization therapy (crt), right ventricular time, transvenous pacemaker, pacing-induced cardiomyopathy, leadless pacemaker

## Abstract

Background: Pacing-induced cardiomyopathy (PICM) is a clinical syndrome that is characterized by a drop in the left ventricular ejection fraction (LVEF) due to chronic high-burden right ventricular (RV) pacing. It has been postulated that leadless pacemakers (LPs) cause decreased risk of PICM compared to transvenous pacemakers (TVPs), but the exact risk reduction is unknown.

Methods: We performed a single-center retrospective analysis of adults who received an LP or TVP between January 1, 2014, and April 1, 2022, and had echocardiograms before and after the pacemaker implant. This study’s outcomes were the RV pacing percentage, change in EF, the need for cardiac resynchronization therapy (CRT) upgrade, and follow-up duration. A Wilcoxon rank-sum test calculated the change in EF. RV time, defined as the duration from pacemaker placement to the follow-up echocardiogram in months multiplied by the RV pacing percentage, served as a surrogate for how long the RV was paced.

Results: A total of 614 patients were screened, and 198 patients were included in the study, where 72 received LP and 126 received TVP. The median follow-up was 480 days. The average of the reported RV percentage pacing was 63.43% for LP and 71.30% for TVP (p=0.14). The incidence of PICM and CRT upgrade was 44% and 9.7% in the LP group and 37% and 9.5% in the TVP group (p=0.3 and p>0.9), respectively. After accounting for age, sex, LP versus TVP, atrioventricular nodal ablation, RV pacing percentage, and follow-up duration, univariate analysis showed that RV time was significantly different between the two types of pacemakers (13.54 ± 14.21 months (LP) versus 9.26 ± 13.95 months (TVP), p=0.009). The difference in RV time between patients who underwent CRT upgrade and those who did not was statistically insignificant (12.11 ± 14.47 months (no CRT) versus 9.19 ± 12.00 months (CRT), p=0.5).

Conclusions: This analysis demonstrated that the incidence of PICM was high in both groups (44% (LP) versus 37% (TVP)), despite significantly more RV time in patients with LP. There was no difference in CRT upgrade between LP and TVP.

## Introduction

The placement of permanent pacemakers (PPMs) has become widely accepted in the management of bradyarrhythmia and is recommended by the current guidelines for the treatment of high-degree heart blocks and symptomatic bradycardia. In fact, approximately one million PPMs are implanted annually worldwide [1-3]. However, excessive right ventricle (RV) pacing was found to have been associated with the development of left ventricular (LV) systolic dysfunction.

Pacing-induced cardiomyopathy (PICM) is generally defined as a drop in the left ventricular ejection fraction (LVEF) in the setting of chronic, high-burden RV pacing and in the absence of other causes (e.g., ischemia). The incidence rates of PICM that were reported in the literature have ranged from 10% to 26%, mostly due to the different LVEF criteria that were used to establish the diagnosis. One popular and agreed-upon definition, although its use resulted in a higher percentage of patients meeting the definition of PICM, is an absolute reduction in LVEF over 10% regardless of baseline, which occurred in about 20% of patients [4-7].

The pathophysiological mechanism underlying PICM is explained by RV pacing and the hemodynamic changes that ensue. Similar to the activation pattern seen in left bundle branch block (LBBB), RV pacing results in delayed depolarization of the LV free wall, which results in electrical and mechanical desynchrony. This results in deleterious remodeling effects on the cardiac tissue ultimately reducing LVEF. These effects are highly associated with chronic RV pacing percentages greater than 20%-40% [1,8-10]. Cardiac resynchronization therapy (CRT) with biventricular (BiV) pacing aims to reestablish electrical and mechanical synchrony by mimicking the normal physiological activation pattern and is currently available for the management of PICM [11].

Several observational studies have tried to identify the risk factors and predictors of PICM, and although these studies could not identify a single predictor for the development of PICM, most of these studies have concluded that wide QRS at baseline, paced QRS prolongation, preimplantation LVEF, and burden of RV pacing were predictors of PICM in RV pacing [12-17]. Other predictors that have been identified include younger age and male sex [18,19]. Some studies have suggested the benefit of septal RV pacing compared to apical RV pacing, given that this is typically associated with a more physiological activation pattern and narrower pQRS [17,20,21]. However, this finding could not be confirmed [21-23]. Other studies have suggested that mechanical complications that are seen more commonly with conventional transvenous pacemakers (TVPs) due to lead placement in the RV apex, such as tricuspid regurgitation (TR), could contribute to the development of PICM. However, the data provided by these studies are conflicting, and the impact of TR in PICM remains unclear [15,24]. Leadless pacemakers (LPs), which offer an alternative to TVPs in patients needing right ventricular pacing, are preferentially implanted in the RV septum and are associated with decreased lead-related complications [17,25].

To date, only two studies have compared the effects of LP and TVP on LVEF. Sanchez et al. [17] conducted an observational study that showed a significantly reduced incidence of PICM in patients with LPs (3%) compared to TVPs (13.7%) in patients with concurrent atrioventricular node (AVN) ablation. Duchenne et al. [25] included 51 patients with an indication for PPM placement, who were randomized to undergo implantation of either LP or TVP. Their prospective analysis concluded that LV function decrease was similar between both groups after 12 months. Our study aimed to compare the incidence of PICM and the need for CRT in patients with LP and TVP.

## Materials and methods

This study was approved by the Institutional Review Board at Michigan State University (approval number: 2136), and data were obtained from a single tertiary center in Michigan, United States. Our analysis reviewed 614 patients and included 198 after using the following inclusion and exclusion criteria: patients 18 years old and older, who received an LP or TVP for the first time between January 1, 2014, and April 1, 2022, and had transthoracic or transesophageal echocardiograms performed before and after the pacemaker implantation. Patients were excluded if they had already had a pacemaker implanted before January 1, 2014, had a BiV pacemaker at the time of initial implant, lacked a baseline or post-pacemaker LVEF evaluation via echocardiogram, or if they lacked right ventricular pacing burden information.

The outcomes of our analysis were RV pacing percentage, change in LVEF, the incidence of PICM, the need for an upgrade to CRT, and RV time. RV pacing percentage was obtained from device interrogation during the first follow-up visit after pacemaker implantation. LVEF was measured, once before implantation and another time at least one month after, using a standard transthoracic or transesophageal echocardiographic technique and analyzed by a qualified experienced cardiologist. The change in LVEF was calculated using the Wilcoxon rank-sum test. PICM was defined as a 10% or greater drop in LVEF on post-pacemaker echocardiogram regardless of baseline and regardless of the presence of heart failure symptoms or signs. The incidence of upgrade to CRT from an RV pacing system was calculated in each group, and so was the duration between the time of initial implant and the time of upgrade. RV time was calculated by multiplying the duration from pacemaker placement to the follow-up echocardiogram in months by the RV pacing percentage and was used to estimate how long the RV was paced in each patient.

Baseline clinical data and procedural characteristics at the time of pacemaker implantation were collected, including patient demographic characteristics (e.g., age and sex), pacemaker indications, the concurrent performance of AVN ablation, pacemaker position (e.g., RV apex and RV septum), and pacemaker type. Preimplant atrioventricular block (AVB) included second‐degree (type I and type II) and complete heart block/high‐grade AVB. Guideline‐directed indications for pacemaker implantation were followed, and the choice of LP versus TVP was based on the operator’s discretion [26].

After accounting for age, sex, LP versus TVP, AVN ablation, RV pacing percentage, and follow-up duration, univariate analysis was done to evaluate the outcomes of the study. Categorical data are represented as numbers and percentages. Continuous data are represented as means and standard deviations. Pearson’s Chi-squared test, Fisher’s exact test, and Wilcoxon rank-sum test were used to analyze the different types of data in this study using the R software. A p-value ≤ 0.05 was considered significant. Kaplan-Meier survival curves were utilized to represent freedom from PICM and CRT between the two groups of the study.

## Results

Of the 614 initially screened patients, 198 patients met the inclusion criteria and were included in our final analysis. The other 416 patients were excluded due to missing data, such as echocardiograms or pacemaker device interrogation, or initial PPM implantation before January 1, 2014. Of our patients, 72 underwent LP placement and 126 received TVP. The baseline and procedural characteristics of the included patients demonstrated that they are similar, except for some differences. Notably, the TVP group had a higher percentage of females and a higher median age (Table 1). The median weighted follow-up period was 480 days.

**Table 1:** Baseline and procedural characteristics of the included patients Values are depicted as numbers (percentages) or means ± standard deviations. AV, atrioventricular; echo, echocardiogram; LP, leadless pacemaker; PPM, permanent pacemaker; RV, right ventricle; TVP, transvenous pacemaker

​​	LP​​	TVP​​
Number of patients​​	72 (36)​​	126 (64)​​
Age (years)​​	73.53 ± 11.90​​​​	79.79 ± 8.58​​
Males​​	52 (72)​​	73 (58)​​
Diagnosis​​	​​	​​
Sick sinus syndrome​​	32 (44)​​	45 (36)​​
Symptomatic bradycardia​​	6 (8)​​	22 (17)​​
AV block	17 (24)​​	28 (22)​​
Tachy-brady syndrome​​	17 (24)​​	31 (25)​​
Pacemaker position​​	​​	​​
RV apex​​	21 (29)​​	97 (77)​​
RV septum​​	50 (69)​​	21 (17)​​
AV nodal ablation​​	9 (12)​​	16 (13)​​
Implant to post-PPM echo (months)​​	15.63 ± 18.13	16.00 ± 14.77

**Figure 1:**
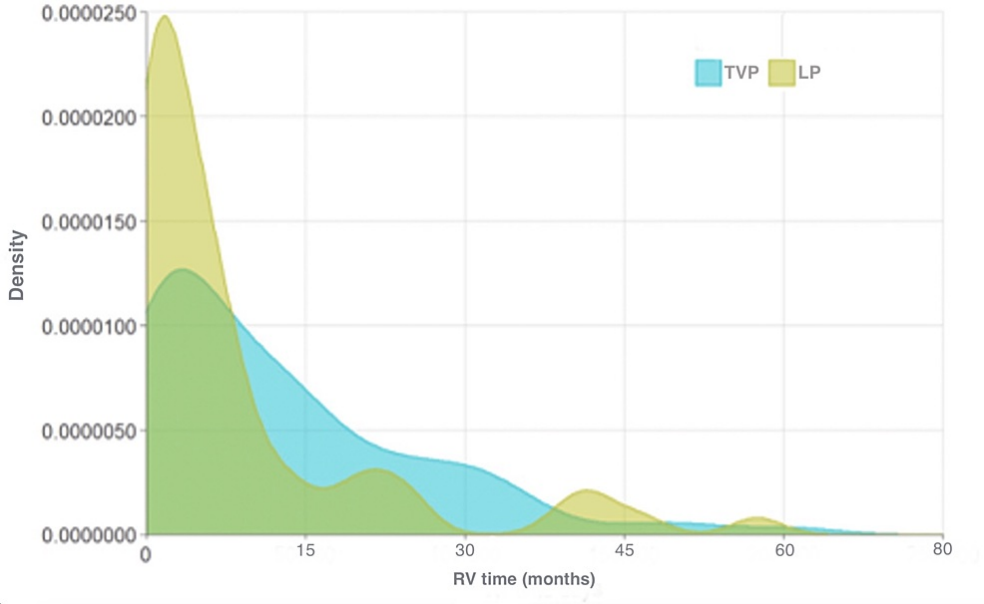
Density plot of RV time based on LP versus TVP RV, right ventricle; LP, leadless pacemaker; TVP, transvenous pacemaker

**Figure 2:**
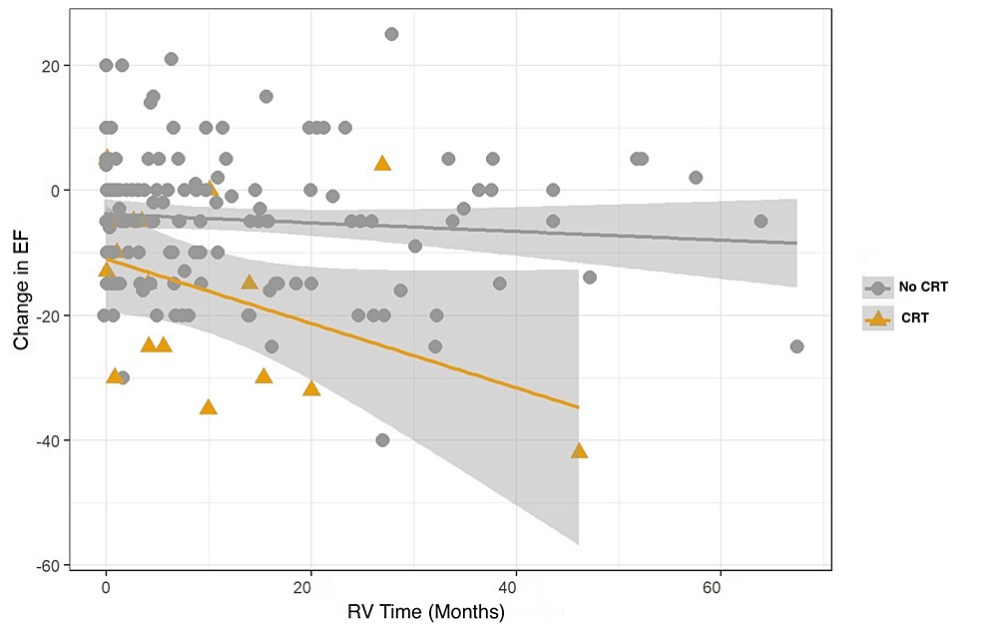
Linear regression between change in EF and RV time in patients who required CRT upgrade and those who did not CRT, cardiac resynchronization therapy; EF, ejection fraction; RV, right ventricle

Univariate analysis showed that RV time was significantly different between pacemakers (13.54 ± 14.21 months (LP) versus 9.26 ± 13.95 months (TVP), p=0.009), but not between patients who required CRT upgrade and those who did not (12.11 ± 14.47 months (no CRT) versus 9.19 ± 12.00 months (CRT), p=0.5) (Table 2 and Table 3, and Figure 1 and Figure 2).

**Table 2:** Univariate analysis evaluating data based on follow-up outcomes Values are depicted as numbers (percentages) or means ± standard deviations. CRT, cardiac resynchronization therapy; LP, leadless pacemaker; LVEF, left ventricle ejection fraction; PICM, pacing-induced cardiomyopathy; RV, right ventricle; TVP, transvenous pacemaker

​	LP​	TVP	P-value​
Change in LVEF	-6.86 ± 12.69	-5.33 ± 11.13	0.2​
PICM​ incidence	32 (44%)​	46 (37%)​	0.3​
RV​ pacing percentage	63.43 ± 38.58	71.30 ± 35.64	0.14​
RV time (months)​	13.54 ± 14.21	9.26 ± 13.95	0.009​
CRT upgrade​	7 (9.7%)​	12 (9.5%)​	>0.9​
Implant to CRT (months)​	12.40 ± 16.33	12.14 ± 14.26	>0.9​

**Table 3:** Univariate analysis evaluating data of patients who received CRT upgrade based on follow-up outcomes Values are means ± standard deviations. CRT, cardiac resynchronization therapy; LVEF, left ventricle ejection fraction; RV, right ventricle

​	CRT upgrade​	No CRT upgrade​	P-value​
LVEF change​	-16.74 ± 14.58 ​	-4.74 ± 10.79	<0.001​
​RV pacing percentage	84.18 ± 27.24​	66.05 ± 37.60	0.11​
RV time (months)​	9.19 ± 12.00	12.11 ± 14.47	0.5​

**Figure 3:**
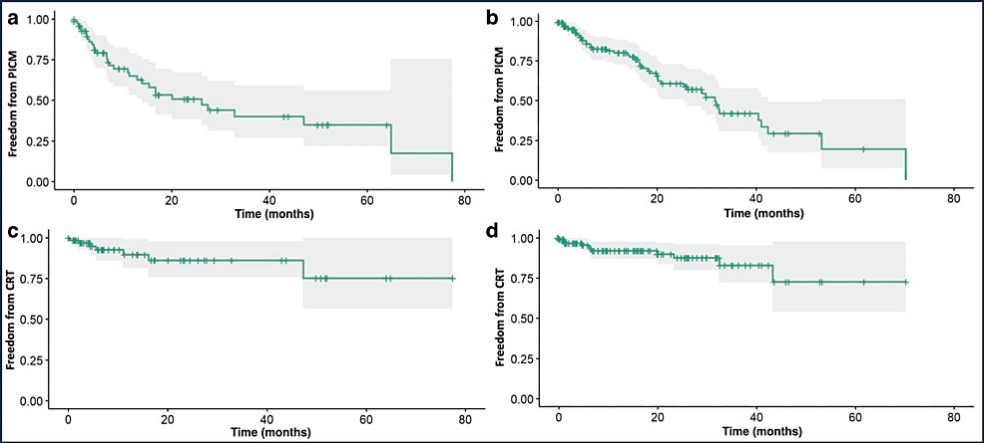
Kaplan-Meier curves Freedom from PICM in LP (a), freedom from PICM in TVP (b), freedom from CRT in LP (c), and freedom from CRT in TVP (d). CRT, cardiac resynchronization therapy; LP, leadless pacemakers; PICM, pacing-induced cardiomyopathy; TVP, transvenous pacemakers

The average RV pacing percentage was found to be 63.43% with LP and 71.30% with TVP (p=0.14). The incidence of PICM and CRT upgrade was 44% and 9.7% in the LP group and 37% and 9.5% in TVP group (p=0.3 and p>0.9), respectively (Table 2). There was no significant difference in the time to develop PICM or time to CRT in patients who required an upgrade between the two groups (Figure 3).

The change in LVEF based on pacer location was different in patients who required CRT upgrade (-8.6 ± 4.48 (RV apex), p=0.07, versus -16.4 ± 6.27 (RV septum), p=0.02) and patients who did not (-4.60 ± 1.46 (RV apex), p=0.0019, versus -2.50 ± 1.61 (RV septum), p=0.12) (Table 4).

**Table 4:** Relation between change in LVEF and pacemaker position in patients with and without CRT CRT, cardiac resynchronization therapy; LVEF, left ventricle ejection fraction; RV, right ventricle

	Change in LVEF	P-value
Without CRT		
RV apex	-4.60 ± 1.46	0.0019
RV septum	-2.50 ± 1.61	0.12
With CRT		
RV apex	-8.6 ± 4.48	0.07
RV septum	-16.4 ± 6.27	0.02

## Discussion

Our analysis found a high incidence of PICM in both LP (44%) and TVP (37%) in patients who required pacemaker placement without a statistically significant difference between the two groups. This is similar to the recently published study by Duchenne et al. [25], which found that the decrease in LVEF was similar in LP and TVP. However, our findings are inconsistent with the data previously published by Sanchez et al. [17], as they reported a lower overall incidence of PICM and reported it to be significantly lower in the LP group (3%) compared to the TVP group (13.7%).

Several factors, mostly related to the differences in the design of the studies, could be responsible for this discrepancy. In Sanchez et al. [17], all the patients who underwent TVP placement and 70% of those who underwent LP placement received concurrent AVN ablation. On the other hand, only a small percentage of our patient population had concurrent AVN ablation during their PPM placement (12% (LP) versus 13% (TVP)). While having concurrent AVN ablation is theoretically expected to result in a higher percentage of RV pacing, the clinical effects of this procedure on developing PICM are still unexplored and warrant further investigation, especially considering these findings.

Additionally, Sanchez et al. [17] included patients who were always pacer dependent and excluded any patient with a percentage RV pacing less than 100%, while our analysis did not have an exclusion criterion related to the level of percentage RV pacing. The average percentage RV pacing was 63.43% ± 38.58% in LP and 71.30% ± 35.64% in TVP. Nonetheless, the incidence of PICM was much higher in our group despite a shorter median follow-up (468.99 ± 543.96 days in LP and 480.44 ± 443.00 days in TVP) and a lower median percentage RV pacing. This indicates that factors other than high-burden RV pacing should be explored for their potential role in developing PICM. Some of the previously proposed factors include baseline LVEF, age, sex, native QRS/paced QRS durations, location of LP pacemaker or pacing leads, and tricuspid regurgitation. Yet, further studies with a larger number of patients are still warranted to delineate the relationship and validate the association between these factors and PICM [12-15,20-23].

In our analysis, despite the lack of significant difference in percentage RV pacing, PICM, and CRT upgrade between both the LP and TVP groups, patients who underwent LP placement had a significantly longer RV time (13.54 ± 14.21 months (LP) versus 9.26 ± 13.95 months (TVP), p=0.009). RV time was statistically insignificant when comparing patients who underwent CRT and those who did not (12.11 ± 14.47 months (no CRT) versus 9.19 ± 12.00 months (CRT), p=0.5). RV time was observed to vary widely between individual patients in our analysis.

The results of our analysis, which showed similar incidence rates of PICM between the two types of PPMs despite a significantly longer RV time in the leadless type, indicate that the degree of RV pacing is not the only predictor of PICM. They also imply that there are differences between LP and TVP that variably affect the LVEF and contribute to the development of PICM. These differences and their effects need further investigation.

Density plots showed significantly higher concentrations of patients with relatively shorter RV times, especially in the LP group (Figure 1). This reflects that only very few patients in this analysis were always pacer dependent, which further supports our claim that factors other than the degree of RV pacing should be evaluated for their potential role in developing PICM.

As expected by the design of the study, which included patients with different LVEF at baseline, and because the majority of patients in both groups had a normal LVEF prior to PPM placement (55.19% ± 8.76% (TVP) and 56.57% ± 9.31% (LP)), only a small percentage of them required upgrade to CRT. Those who did have a significantly reduced LVEF from baseline (-16.74 ± 14.58) compared to those who did not (-4.74 ± 10.79) (p<0.001) (Table 3 and Figure 2).

Some studies have previously suggested a potential benefit to septal pacing, given its more physiologic pattern of ventricular activation [15,18,23]. In this cohort, the majority of the patients in the LP group had the pacer implanted in the RV septum, while most patients in the TVP group had the pacer lead placed in the RV apex. Considering the similar rates of PICM in our study, despite a longer RV time and mostly septal placement of LP, we compared the outcomes based on septal versus apical pacer location. Similar to the data from previous studies, the differences were insignificant [18,23].

In fact, upon further analysis, the relation between pacer location and change in LVEF among patients who underwent CRT upgrade showed that the septal position was associated with an average drop of 16% (p=0.02). Apical placement, on the other hand, was associated with an average drop equal to 8.6% (p=0.07). A similar analysis of patients with PICM, who did not have CRT upgrade, showed that apical pacing was associated with a 5% drop in LVEF (p=0.0019), while the results for septal pacing were statistically insignificant (Table 4).

There are limitations to this study that need to be acknowledged. This analysis is retrospective in nature with a small number of patients from a single center. Additionally, our definition of PICM, similar to previous studies, was based only on the change in LVEF without any clinical criteria. Moreover, based on time constraints, we did not record EF and RV pacing percentages on subsequent echocardiograms and pacemaker device interrogations. Finally, only a small subset of our patient population underwent concurrent AVN ablation, which makes it challenging to directly compare our results with those of Sanchez et al. [17].

## Conclusions

Our analysis demonstrated that despite longer RV time with LP compared to TVP, the incidence of PICM was high in both types of PPM without a significant difference. RV time was statistically insignificant in predicting the need for a CRT upgrade, and pacer location did not show significant differences in the incidence of PICM or the need for a CRT upgrade. Further studies with a larger number of patients are warranted to further explore the differences between these types of PPM, as well as consider other factors that could contribute to PICM.

## References

[1] Abbas J, Zulqarnain M, Waqar F, Waqar Z, Malik J, Satti DI, Zaidi SM (2022). Incidence and predictors of pacemaker-induced cardiomyopathy with right ventricular pacing: a systematic review. Expert Rev Cardiovasc Ther.

[2] Nielsen JC, Kristensen L, Andersen HR, Mortensen PT, Pedersen OL, Pedersen AK (2003). A randomized comparison of atrial and dual-chamber pacing in 177 consecutive patients with sick sinus syndrome: echocardiographic and clinical outcome. J Am Coll Cardiol.

[3] Abdin A, Yalin K, Zink MD, Napp A, Gramlich M, Marx N, Schuett K (2019). Incidence and predictors of pacemaker induced cardiomyopathy: a single-center experience. J Electrocardiol.

[4] Merchant FM (2019). Pacing-induced cardiomyopathy: just the tip of the iceberg?. Eur Heart J.

[5] Merchant FM, Mittal S (2018). Pacing-induced cardiomyopathy. Card Electrophysiol Clin.

[6] Khurshid S, Epstein AE, Verdino RJ, Lin D, Goldberg LR, Marchlinski FE, Frankel DS (2014). Incidence and predictors of right ventricular pacing-induced cardiomyopathy. Heart Rhythm.

[7] Gavaghan C (2022). Pacemaker induced cardiomyopathy: an overview of current literature. Curr Cardiol Rev.

[8] Dreger H, Maethner K, Bondke H, Baumann G, Melzer C (2012). Pacing-induced cardiomyopathy in patients with right ventricular stimulation for >15 years. Europace.

[9] Ghani A, Delnoy PP, Ottervanger JP, Ramdat Misier AR, Smit JJ, Elvan A (2011). Assessment of left ventricular dyssynchrony in pacing-induced left bundle branch block compared with intrinsic left bundle branch block. Europace.

[10] Sweeney MO, Hellkamp AS, Ellenbogen KA, Greenspon AJ, Freedman RA, Lee KL, Lamas GA (2003). Adverse effect of ventricular pacing on heart failure and atrial fibrillation among patients with normal baseline QRS duration in a clinical trial of pacemaker therapy for sinus node dysfunction. Circulation.

[11] Guglin M, Barold SS (2015). The role of biventricular pacing in the prevention and therapy of pacemaker-induced cardiomyopathy. Ann Noninvasive Electrocardiol.

[12] Laksono S, Setianto B, Iqbal M, Prawara AS (2022). Understanding pacemaker-induced cardiomyopathy incidence and predictors in patients with right ventricular pacing: a systematic review. Int J Angiol.

[13] Kim JH, Kang KW, Chin JY, Kim TS, Park JH, Choi YJ (2018). Major determinant of the occurrence of pacing-induced cardiomyopathy in complete atrioventricular block: a multicentre, retrospective analysis over a 15-year period in South Korea. BMJ Open.

[14] Kiehl EL, Makki T, Kumar R (2016). Incidence and predictors of right ventricular pacing-induced cardiomyopathy in patients with complete atrioventricular block and preserved left ventricular systolic function. Heart Rhythm.

[15] Bansal R, Parakh N, Gupta A (2019). Incidence and predictors of pacemaker-induced cardiomyopathy with comparison between apical and non-apical right ventricular pacing sites. J Interv Card Electrophysiol.

[16] Kaye G, Ng JY, Ahmed S, Valencia D, Harrop D, Ng AC (2019). The prevalence of pacing-induced cardiomyopathy (PICM) in patients with long term right ventricular pacing - is it a matter of definition?. Heart Lung Circ.

[17] Sanchez R, Nadkarni A, Buck B (2021). Incidence of pacing-induced cardiomyopathy in pacemaker-dependent patients is lower with leadless pacemakers compared to transvenous pacemakers. J Cardiovasc Electrophysiol.

[18] Zhang H, Zhou YJ, Zeng YJ (2020). Prognostic factors of pacing-induced cardiomyopathy. Chin Med J (Engl).

[19] Karpawich PP, Mital S (1997). Comparative left ventricular function following atrial, septal, and apical single chamber heart pacing in the young. Pacing Clin Electrophysiol.

[20] Mera F, DeLurgio DB, Patterson RE, Merlino JD, Wade ME, León AR (1999). A comparison of ventricular function during high right ventricular septal and apical pacing after his-bundle ablation for refractory atrial fibrillation. Pacing Clin Electrophysiol.

[21] Kypta A, Steinwender C, Kammler J, Leisch F, Hofmann R (2008). Long-term outcomes in patients with atrioventricular block undergoing septal ventricular lead implantation compared with standard apical pacing. Europace.

[22] Nikoo MH, Ghaedian MM, Kafi M (2011). Effects of right ventricular septal versus apical pacing on plasma natriuretic peptide levels. J Cardiovasc Dis Res.

[23] Victor F, Mabo P, Mansour H (2006). A randomized comparison of permanent septal versus apical right ventricular pacing: short-term results. J Cardiovasc Electrophysiol.

[24] Van De Heyning CM, Elbarasi E, Masiero S (2019). Prospective study of tricuspid regurgitation associated with permanent leads after cardiac rhythm device implantation. Can J Cardiol.

[25] Duchenne J, Garweg C, Puvrez A, Mao Y, Ector J, Willems R, Voigt JU (2022). The effect of leadless pacing on LV and RV systolic function is not inferior to conventional RV pacing. Eur Heart J.

[26] Wilkoff BL, Auricchio A, Brugada J (2008). HRS/EHRA Expert Consensus on the Monitoring of Cardiovascular Implantable Electronic Devices (CIEDs): description of techniques, indications, personnel, frequency and ethical considerations: developed in partnership with the Heart Rhythm Society (HRS) and the European Heart Rhythm Association (EHRA); and in collaboration with the American College of Cardiology (ACC), the American Heart Association (AHA), the European Society of Cardiology (ESC), the Heart Failure Association of ESC (HFA), and the Heart Failure Society of America (HFSA). Endorsed by the Heart Rhythm Society, the European Heart Rhythm Association (a registered branch of the ESC), the American College of Cardiology, the American Heart Association. Europace.

